# Clinical Application of Artificial Intelligence in the Ultrasound Classification of Hepatic Cystic Echinococcosis

**DOI:** 10.4269/ajtmh.23-0519

**Published:** 2024-05-28

**Authors:** Feng Shang, Tao Song, Zhengye Wang, Miao Wu, Chuanbo Yan, Xiaorong Wang

**Affiliations:** ^1^ State Key Laboratory of Pathogenesis, Prevention and Treatment of High Incidence Diseases in Central Asia, Ultrasonography Department, The First Affiliated Hospital of Xinjiang Medical University, Urumqi, Xinjiang Uygur Autonomous Region, China;; ^2^ State Key Laboratory of Pathogenesis, Prevention and Treatment of High Incidence Diseases in Central Asia, College of Medical Engineering Technology, Xinjiang Medical University, Urumqi, Xinjiang Uygur Autonomous Region, China

## Abstract

Hepatic cystic echinococcosis (HCE) is a zoonotic disease that occurs when the larvae of *Echinococcus granulosus* parasitize the livers of humans and mammals. HCE has five subtypes, and accurate subtype classification is critical for choosing a treatment strategy. To evaluate the clinical utility of artificial intelligence (AI) based on convolutional neural networks (CNNs) in the classification of HCE subtypes via ultrasound imaging, we collected ultrasound images from 4,012 HCE patients at the First Affiliated Hospital of Xinjiang Medical University between 2008 and 2020. Specifically, 1,820 HCE images from 967 patients were used as the training and validation sets for the construction of the AI model, and the remaining 6,808 images from 3,045 patients were used as the test set to evaluate the performance of the AI models. The 6,808 images were randomly divided into six groups, and each group contained equal proportions of the five subtypes. The data of each group were analyzed by a resident physician. The accuracy of HCE subtype classification by the AI model and by manual inspection was compared. The AI HCE classification model showed good performance in the diagnosis of subtypes CE1, CE2, CE4, and CE5. The overall accuracy of the AI classification (90.4%) was significantly greater than that of manual classification by physicians (86.1%; *P* <0.05). The CNN can better identify the five subtypes of HCE on ultrasound images and should help doctors with little experience in more accurately diagnosing HCE.

## INTRODUCTION

Cystic echinococcosis (CE) is a zoonotic disease caused by *Echinococcus granulosus* tapeworms in humans and mammals.^1^ The distribution of CE is worldwide, and areas with high incidence include western China, Central Asia, South America, Mediterranean countries, and East Africa.^2^ In endemic areas, the annual incidence of CE ranges from <1 to 200 per 100,000 inhabitants.^3^ CE can occur in all organs but occurs most frequently in the liver, and HCE accounts for approximately 70% of cases.^4^ In 2003, the WHO Informal Working Group on Echinococcosis (IWGE) proposed a standardized ultrasound (US) classification based on the active–transitional–inactive status of the cyst as suggested by its sonographic appearance, which reflects the current knowledge of the natural history of CE.^5^ There are five subtypes of CE according to standardized US classification: single cystic type (CE1), polycystic type (CE2), internal capsule collapse type (CE3), solid type (CE4), and calcification type (CE5). The tapeworms in subtypes CE1 and CE2 are in the growth and development stage and exhibit growth and reproductive activities. Changes to cyst tend toward inactivity in a process that is favorable to the host. CE1 progress through CE3 to CE4. The tapeworms in CE3 are in the transitional stage and have reduced activity, but they still have developmental ability. Reactivation from stage CE3 can produce CE2 cysts.^6^ Three major approaches (drug treatment, surgery, and puncture–aspiration–injection–reaspiration) can be used to treat CE1, CE2, and CE3 according to the type, size, location, number of cysts, and presence of complications.^2^^,^^7^ CE4 and CE5 hydatids lose their ability to develop and are inactive; therefore, they can be treated with a watch-and-wait strategy.^1^ Because the treatments for the different CE subtypes can vary, it is essential to classify CE correctly. However, acceptance of the standardized US classification is rather poor. In 71.2% of the publications, cyst classification was not provided. Among those publications where in classification was conducted, 14% used the WHO-IWGE classification.^8^ Ultrasound is the cornerstone of diagnosis, staging, and follow-up for treating CE cysts and has the advantages of being noninvasive, inexpensive, highly specific, and accurate; additionally, US is superior to computed tomography or magnetic resonance imaging for staging cysts.^9^ However, US is easily affected by operator experience, and the diagnostic capabilities of doctors can markedly differ across regions and hospitals. Most patients with CE are herdsmen in remote areas with limited medical resources. Thus, a convenient, fast, and accurate diagnostic technique will benefit these patients.

The combination of medical imaging and artificial intelligence (AI) was proposed as early as the 1960s.^10^ Deep learning is a subfield of AI, also known as “representation learning,” which performs multilevel learning with cascaded nonlinear modules. Starting from the raw input, each module transforms a representation at one level into a representation at a higher, more abstract level. By combining enough such transformations, complex functions can be learned. For a classification task, the higher level representations amplify the aspects of the input that are most important in distinguishing the classes and suppressing irrelevant changes, and deep learning has a great advantage in such tasks.^11^^,^^12^ A convolutional neural network (CNN) is a representative model of deep learning. Its greatest advantage is that it can automatically detect significant features without any human supervision. It is currently used in the fields of computer vision, speech processing, and facial recognition, among other features.^12^^–^^16^ CNN has achieved human-level performance in several object classification tasks, and it has produced good results in medical image recognition and classification.^12^^,^^17^^–^^21^ It is expected that CNN can be used to classify HCE sonograms to improve the accuracy of HCE classification by radiologists based on ultrasound images.

## MATERIALS AND METHODS

**Figure 1.**
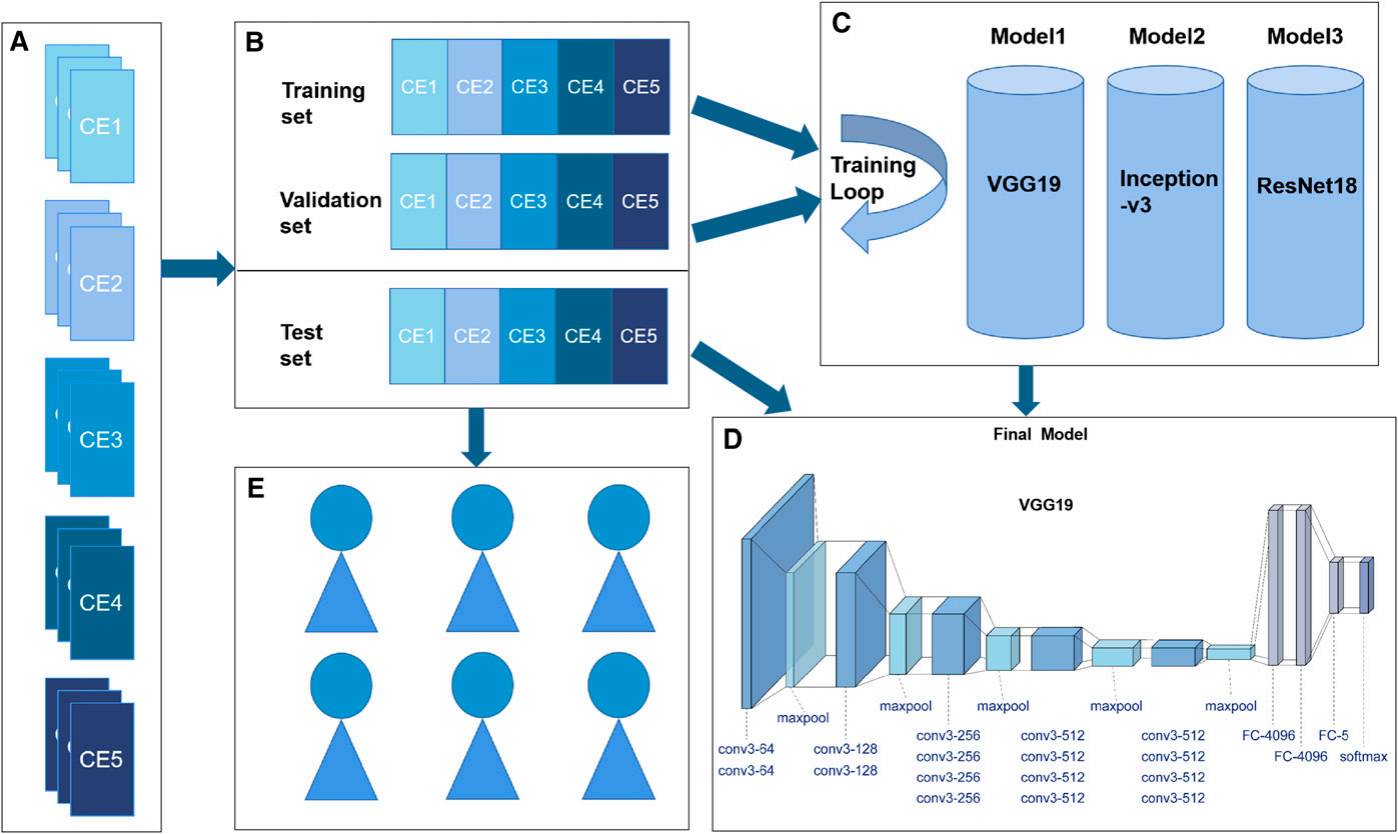
Hepatic cystic echinococcosis (HCE) ultrasound image collection, grouping, convolutional neural network model construction, and final evaluation. (**A**) Collection of ultrasound images of each subtype of HCE. (**B**) The ultrasound images were divided into training, validation, and final test sets. (**C**) Three types of CNN architectures (Visual Geometry Group 19 [VGG19], Inception-v3, and deep residual network 18 [ResNet18]) were trained and validated based on the training set and validation set. (**D**) The final model was evaluated with the test set. (**E**) The images in the test set were evenly assigned to six resident physicians, with equal proportions of the HCE subtypes to be classified.

In this study, we used the following processes: collection of HCE ultrasound images, construction of AI classification models, evaluation of the models, and comparison of AI classification and manual classification using the test set (Figure 1). This study was approved by the Ethics Committee of The First Affiliated Hospital of Xinjiang Medical University, Xinjiang, China.

### Study materials.

**Figure 2.**
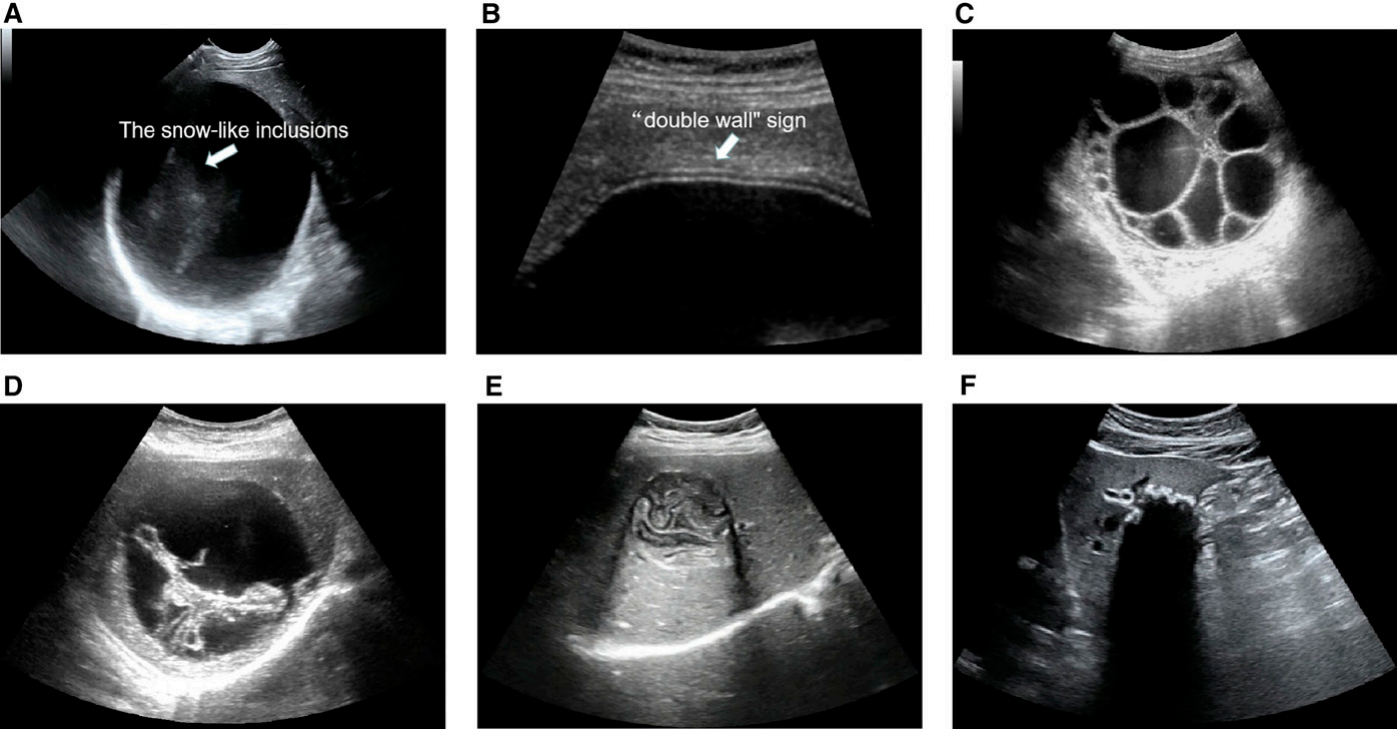
Sonographic images of patients with different hepatic cystic echinococcosis (CE) subtypes. (**A**) Subtype CE1; the arrow indicates snow-like inclusions, which is also known as “hydatid sand.” (**B**) The arrow indicates the “double wall” sign. (**C**) Subtype CE2. (**D**) Subtype CE3. (**E**) Subtype CE4. (**F**) Subtype CE5.

A total of 9,723 US images in JPG format were collected from 4,153 HCE patients between 2008 and 2020. The images were stored in the PACS system of The First Affiliated Hospital of Xinjiang Medical University. These patients were confirmed to have HCE via surgical pathology or long-term follow-up with various imaging techniques. The images were screened by two senior radiologists, and images that were unclear or had incomplete lesions were excluded. Ultimately, 8,628 HCE images from 4,012 patients were included. The two senior radiologists then manually classified these 8,628 images. In cases of disagreement, a senior radiologist with 30 years of experience in the US diagnosis of HCE determined the subtype. After manual classification, 1,821 images from 863 CE1 patients (Figure 2A and B), 2,484 images from 1,012 CE2 patients (Figure 2C), 736 images from 314 CE3 patients (Figure 2D), 2,318 images from 1,082 CE4 patients (Figure 2E), and 1,269 images from 741 CE5 patients (Figure 2F) were included in the subsequent analysis. The sonographic features of each subtype of CE are as follows. Subtype CE1 has a round or oval cystic lesion with a clear boundary and a smooth cyst wall, accompanied by enhanced posterior echoes. There is a potential gap between the inner and outer cyst walls, thus generating a “double-walled” sign. Such cysts may or may not contain small, dense mobile echoes, snowlike inclusions (these are floating brood capsules that are often called hydatid sand, a combination of fluid and proto-scolices). Subtype CE2 has multiple daughter cysts of various sizes in the anechoic area of the parental cyst, thus generating a “cyst in the cyst” sign. When there are few daughter cysts, they are often arranged close to the wall of the parental cyst, whereas if there are many daughter cysts, they may fill the whole parental cyst, thus displaying a “beehive” sign. Subtype CE3 has an inner cyst wall that is partially or completely ruptured and floats in the cystic fluid. Part of the inner wall falls away from the outer wall to display a “sky curtain” sign. The inner wall collapses further and shrinks to show a “drift belt.” Inner wall complete detachment observed by ultrasonography is referred to as the water-lily sign. Subtype CE4 undergoes degeneration and necrolysis. The cyst fluid is absorbed and reduced, and the cyst wall folds and shrinks, thus generating areas of heterogeneous hypoechoic or hyperechoic degeneration on sonographs, may show a “ball of wool” sign or the “cerebral gyrus” sign, which is indicative of degenerating membranes. In subtype CE5, the outer cyst wall of the hydatid is thickened and roughened by calcium salt deposition, and a hyperechoic contour and a wide posterior acoustic shadow can be observed. Partial calcification generates strong arc-shaped echoes, and complete calcification generates “eggshell-like” changes.

### Methods.

The 8,628 HCE ultrasound images from 4,012 patients were randomly divided into a training set + validation set (1,820 images from 967 patients) and a test set (6,808 images from 3,045 patients).

#### Construction of an AI model for the classification of HCE sonograms.

A total of 1,820 HCE ultrasound images from 967 patients were used to train the CNN, including 358 images of CE1-type HCE from 174 patients, 383 images of CE2-type HCE from 194 patients, 350 images of CE3-type HCE from 156 patients, 378 images of CE4-type HCE from 230 patients, and 351 images of CE5-type HCE from 213 patients. The images were first preprocessed, including the removal of artificial marks and the extraction of regions of interest. The Spot Healing Brush of Adobe Photoshop (version 19.1.1; Adobe Systems Incorporated, San Jose, CA) was used to remove any artificial markers or irrelevant information that could affect the classification. The images were repaired to improve the reliability of the models. Lesion areas were manually cropped as regions of interest and converted to the JPG format to a size of 224 × 224 pixels. Three CNN architectures, including Visual Geometry Group 19 (VGG19),^22^ Inception-v3,^23^ and deep residual network 18 (ResNet18)^24^ (which perform well in image recognition), were selected for model construction. Preprocessed images were used to train the models. Each of these three models has two scenarios: 1) networks with random initial weights and 2) pretrained networks with publicly available weights. The initial learning rate was set to 0.0003, and 5-fold cross-validation was repeated five times. In each of the 5-fold cross-validation procedures, the 1,820 images were split into five groups; in each repeat, four groups were combined as the training set, and the remaining group was used for validation. These models were trained with MATLAB 2020b software on a computer with 32 GB of memory and an Intel^®^ Core™ i7-8700 at 3.2 GHz CPU. The VGG19 model that was pretrained with publicly available weights achieved the highest accuracy (90.6%) in classifying the HCE ultrasound images. A desktop application for the classification of HCE based on VGG19 was subsequently developed. After the HCE image to be identified was selected and the lesion was manually framed, the probability of each of the five HCE subtypes was calculated. A higher probability would more likely indicate that HCE was the corresponding subtype.

#### Comparison of AI classification and manual classification in the test set.

The 6,808 HCE ultrasound images from 3,045 patients in the test set were used to compare the accuracy of the AI classification and manual classification by resident physicians. The test set included 1,463 images of CE1-type HCE from 689 patients, 2,101 images of CE2-type HCE from 818 patients, 386 images of CE3-type HCE from 158 patients, 1,940 images of CE4-type HCE from 852 patients, and 918 images of CE5-type HCE from 528 patients.

An attending physician input the images into the AI software for classification and recorded the results. The 6,808 images were randomly divided into six groups, each of which contained equal proportions of the five subtypes. Each of six physicians randomly selected one group, and the images were manually classified. The classification results of the six residents were summarized.

## STATISTICAL ANALYSES

The precision, recall, specificity, and F1 score (the harmonic mean of precision and recall) were used to evaluate the performance of the VGG19-based AI software in classifying ultrasound images from HCE patients. A paired χ^2^ test was used to compare the accuracy of the AI classification and the manual classification by the residents. All statistical analyses were performed with SPSS 26 software, and differences were considered statistically significant at two-sided *P* <0.05.

## RESULTS

Performance of the VGG19-based AI model in identifying HCE subtypes (Table 1). Visual Geometry Group 19 had relatively poor precision and a low F1 score for detecting CE3-type HCE and showed suboptimal recognition of this subtype. However, its precision and recall rates for the other four subtypes were >0.85, and the F1 scores were >0.90, indicating good recognition ability.

**Table 1:** Performance of the VGG19-based AI model in identifying HCE subtypes

HCE Subtypes	Precision	Recall	Specificity	F1-Score
CE1	0.947	0.887	0.987	0.916
CE2	0.980	0.853	0.992	0.912
CE3	0.577	0.902	0.960	0.704
CE4	0.930	0.939	0.971	0.934
CE5	0.854	0.974	0.974	0.910

AI = artificial intelligence; CE = cystic echinococcosis; HCE = hepatic cystic echinococcosis; VGG19 = Visual Geometry Group 19.

### Artificial intelligence and manual classification of HCE subtypes.

In the test set, the AI (VGG19) correctly classified 6,153 of the total 6,808 images, thus representing 90.4% accuracy, and the physicians correctly classified 5,861 images (86.1% accuracy). Thus, the AI was significantly more accurate than the physicians in classifying the HCE subtypes (*P* <0.05; Table 2).

**Table 2:** Classification of HCE ultrasound images by AI and physicians (number/%)

HCE Subtypes	AI (VGG19)		Resident Physician		*P*-Value
	Exactness	Mistake	Exactness	Mistake	
CE1 (1463)	1,297 (88.7)	166 (11.3)	1,334 (91.2)	129 (8.8)	0.028
CE2 (2101)	1,792 (85.3)	309 (14.7)	1,706 (81.2)	395 (18.8)	<0.001
CE3 (386)	348 (90.2)	38 (9.8)	328 (85.0)	58 (15.0)	0.029
CE4 (1940)	1,822 (93.9)	118 (6.1)	1,689 (87.1)	251 (12.9)	<0.001
CE5 (918)	894 (97.4)	24 (2.6)	804 (87.6)	114 (12.4)	<0.001
CE1–CE5 (6808)	6,153 (90.4)	655 (9.6)	5,861 (86.1)	947 (13.9)	<0.001

AI = artificial intelligence; CE = cystic echinococcosis; HCE = hepatic cystic echinococcosis; VGG19 = Visual Geometry Group 19.

The recall of AI in identifying CE1- and CE2-type HCE was <90% in both cases, and the recall was lowest for CE2-type HCE (85.3%). The recall of AI in classifying the other three subtypes was >90% in all cases and was highest for CE5-type HCE cases (97.4%). The recall of the physicians in classifying CE1-type HCE was >90%, which was significantly greater than that of the AI (*P* <0.05). However, for the other four subtypes, the recall of physicians was <90% in all cases, which was also significantly lower than that of the AI (*P* <0.05). Subtype CE2 had the lowest recall of classification by both the AI and physicians (85.3% and 81.2%, respectively); however, the AI still performed better than the physicians on this subtype (*P* <0.05).

**Figure 3.**
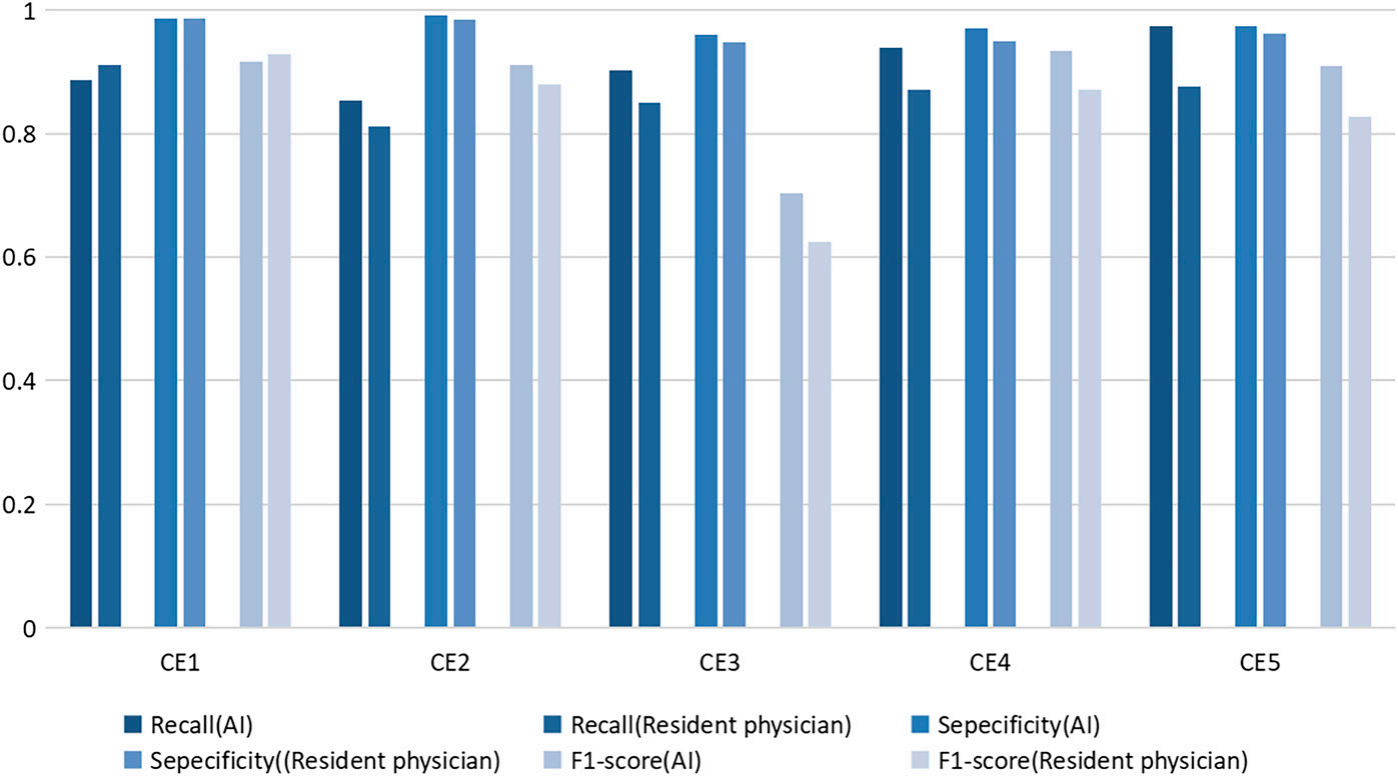
The recall, specificity, and F1-score of the artificial intelligence and resident in classifying the hepatic cystic echinococcosis (CE) subtypes.

We compared the recall, specificity, and F1-score between the AI and resident in classifying the HCE subtypes (Figure 3). The AI had lower recall and F1-scores for identifying CE1 than did residents, as well as a slightly greater specificity than did residents. The AI identified CE2 through CE5 more accurately than did the residents, with higher recall, specificity, and F1-scores.

Patients with CE1-, CE2-, or CE3-type HCE require further treatment, whereas follow-up observation is generally recommended for those with CE4- or CE5-type HCE. Therefore, types CE1, CE2, and CE3 are collectively referred to as “treatment-required HCE,” whereas CE4 and CE5 are collectively referred to as “treatment-free HCE.” A comparison of the sensitivity of the AI and physician methods for identifying treatment-required HCE (CE1–CE3) and treatment-free HCE (CE4 and CE5) is shown in Table 3.

**Table 3:** Identification of treatment-required HCE and treatment-free HCE by AI and the physicians (number/%)

Actual HCE Subtypes	AI		Resident Physician		*P*-Value
	Treatment-Required HCE	Treatment-Free HCE	Treatment-Required HCE	Treatment-Free HCE	
Treatment required HCE (3950)	3,774 (95.5)	176 (4.5)	3,772 (95.5)	178 (4.5)	0.924
Treatment free HCE (2858)	27 (0.9)	2,831 (99.1)	76 (2.7)	2,782 (97.3)	<0.001

AI = artificial intelligence; HCE = hepatic cystic echinococcosis.

Both the AI and the physicians distinguished treatment-required and treatment-free HCE very accurately (all >95%). There was no significant difference between AI and the physicians in identifying treatment-required HCE (both 95.5%); however, AI showed greater sensitivity in identifying treatment-free HCE than did the physicians (99.1% versus 97.3%, respectively; *P* <0.05).

### Misclassification of HCE subtypes by AI (VGG19) and physicians.

The HCE subtypes that were misclassified by AI and the physicians are shown in Table 4.

**Table 4:** HCE subtypes misclassified by AI and resident physicians, number (%)

Actual HCE Subtypes	Misclassified HCE Subtypes					
		CE1	CE2	CE3	CE4	CE5
CE1	AI (166)	–	14 (8.4)	122 (73.5)	5 (3.0)	25 (15.1)
	RP (129)	–	39 (30.2)	73 (56.6)	15 (11.6)	2 (1.6)
CE2	AI (309)	64 (20.7)	–	110 (35.6)	103 (33.3)	32 (10.4)
	RP (395)	56 (14.2)	–	204 (51.6)	119 (30.1)	16 (4.1)
CE3	AI (38)	7 (18.4)	20 (52.6)	–	9 (23.7)	2 (5.3)
	RP (58)	12 (20.7)	20 (34.5)	–	19 (32.7)	7 (12.1)
CE4	AI (118)	1 (0.8)	3 (2.5)	20 (17.0)	–	94 (79.7)
	RP (251)	4 (1.6)	7 (2.8)	43 (17.1)	–	197 (78.5)
CE5	AI (24)	0 (0)	0 (0)	3 (12.5)	21 (87.5)	–
	RP (114)	3 (2.6)	4 (3.5)	15 (13.2)	92 (80.7)	–

AI = artificial intelligence; CE = cystic echinococcosis; HCE = hepatic cystic echinococcosis; RP = resident physicians.

**Figure 4.**
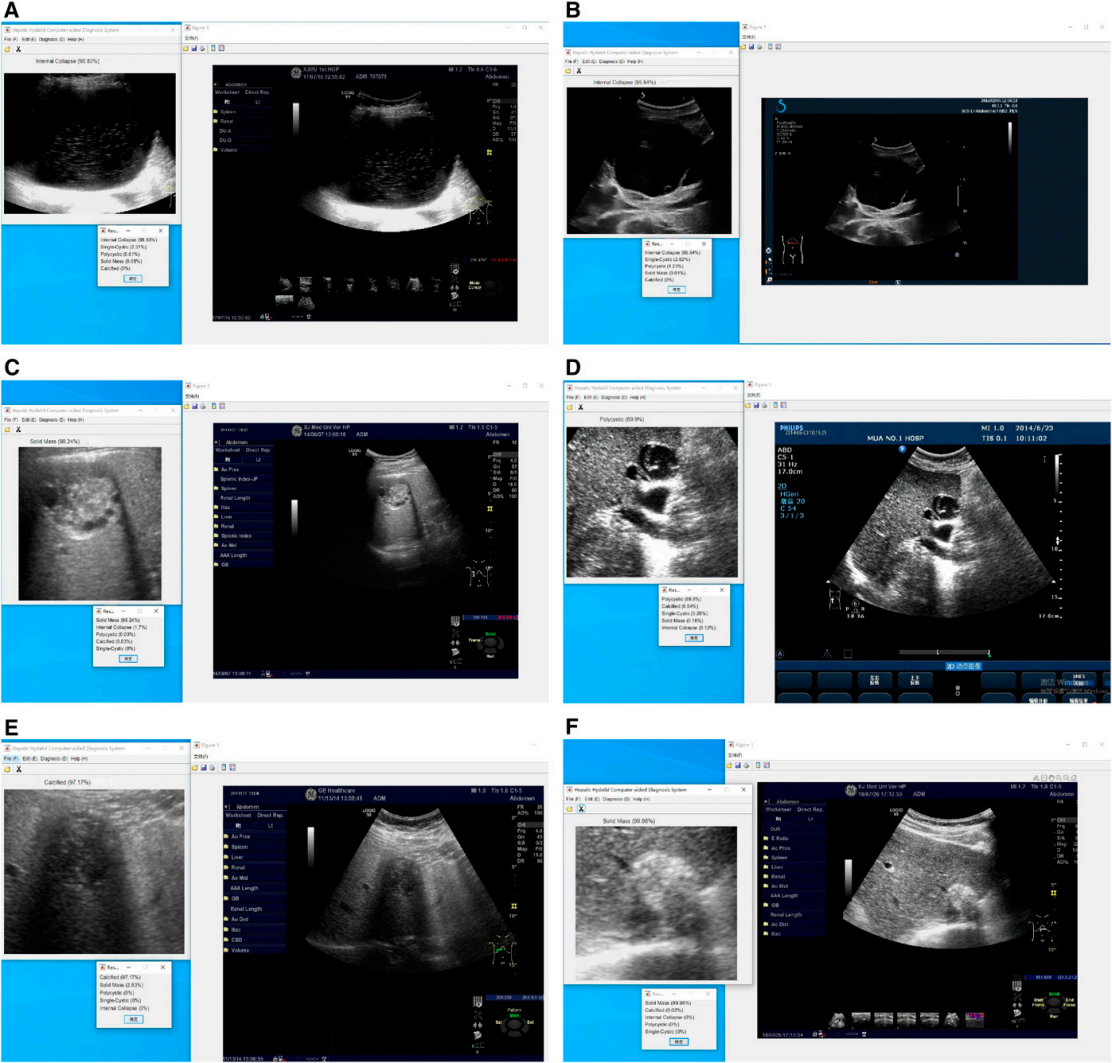
Cases misclassified with Visual Geometry Group 19 artificial intelligence. (**A**) Cystic echinococcosis (CE)1 was misclassified as CE3. (**B**) Subtype CE2 was misclassified as CE3. (**C**) Subtype CE2 was misclassified as CE4. (**D**) Subtype CE3 was misclassified as CE2. (**E**) Subtype CE4 was misclassified as CE5. (**F**) Subtype CE5 was misclassified as CE4.

The AI algorithm was most likely to misclassify CE1 as CE3, and this error accounted for 73.5% of all cases of CE1 misclassification (Figure 4A). CE2 was more likely to be misclassified as CE3 (Figure 4B) or CE4 (Figure 4C) by AI, and these errors accounted for 35.6% and 33.3%, respectively, of all cases of CE2 misclassification. The misclassification of the CE3 subtype as the CE2 subtype accounted for 52.6% of all cases of CE3 misclassification (Figure 4D). Moreover, CE4 was likely to be misclassified as CE5 (79.7%) (Figure 4E), and CE5 was likely to be misclassified as CE4 (87.5%) (Figure 4F).

The most frequent misclassification of the CE1 subtype by both AI and physicians was its identification as CE3. The second most frequent misclassification of subtype CE1 differed between AI and physicians, with CE1 misclassified as CE5 by AI, and CE1 misclassified as CE2 by physicians. In the classification of CE2–CE5, the most frequent and second most frequent misclassified subtypes were the same for the AI and the physicians.

## DISCUSSION

Cystic echinococcosis is highly prevalent in pastoral areas; moreover, with the development of global tourism, this condition is globally distributed. HCE is a major public health problem and an economic burden in many areas, especially those with low levels of economic development.^25^^,^^26^ The impact of CE can be presented in both nonmonetary and monetary terms. In terms of monetary burden, based on a 2006 estimate of the global burden of CE, monetary annual losses attributable to human CE were estimated to be $193 million and increased to $764 million when adjusted for underreporting.^27^ In terms of nonmonetary burden, current estimates of the global burden average 285,500 disability-adjusted life years (DALYs) for human CE (5–7) (>1 million if underreporting is taken into account).^1^ China is one of the most important endemic areas of CE. The endemic area of CE in China is approximately 4.2 million km^2^, accounting for 44% of the total land area. The areas with the highest incidence are concentrated in the pastoral, semiagricultural, and semipastoral areas in northwestern China. Xinjiang is also a high-incidence area, where approximately 70% of CE patients are children, adolescents, or young adults.^28^^–^^30^ Most HCE patients are asymptomatic in the early stage. As the disease progresses, symptoms and signs may appear, including liver enlargement, right upper quadrant pain, nausea, and vomiting. HCE can lead to a series of severe complications, including hepatic hydatid infection, portal vein or bile duct obstruction, portal hypertension, hepatic hydatid–biliary fistula, cyst leakage or rupture leading to panperitonitis, and systemic allergic reactions.^1^^,^^31^^–^^33^ Ultrasound can detect small HCE cysts at an early stage, and early diagnosis and treatment can reduce the probability of complications and improve patient prognosis. The WHO-IWGE developed a standardized classification of CE based on ultrasonography and introduced a structured stage-specific approach to CE management. Treatment of HCE varies across the five subtypes. Patients in the CE1 to CE3 cohorts required active treatment based on the specific characteristics of the disease and the clinical condition of the patient, whereas patients in the CE4 and CE5 cohorts only required follow-up observation. Therefore, accurate classification of HCE US images is extremely important when choosing a treatment plan.

Compared with human observations, AI-enhanced visual recognition has a lower error rate, which has encouraged the development of AI in medical imaging.^34^^,^^35^ The applications of machine learning to medical imaging include lesion detection and classification, automatic image segmentation, and data analysis.^12^^,^^17^^,^^34^ Deep learning and CNNs have shown excellent performance in image recognition and classification. However, because researchers do not know the features that are important for classification and prediction and cannot explain the process of output generation, deep learning is considered to be a black box.^36^^–^^38^ Several successful studies have evaluated the application of CNNs to disease diagnosis and classification based on medical images. For example, the study by Roblot et al.^39^ showed that an algorithm based on a fast-region CNN could detect a meniscus tear in magnetic resonance images of the knee, with a final weighted area under the curve (AUC) of 0.90. The deep learning model developed by Herent et al.^40^ recognized and classified breast lesions on magnetic resonance images, with a weighted average AUC of 0.82. Moreover, the deep learning model constructed by Schmauch et al. detected and classified focal liver lesions, with a weighted average receiver operating characteristic AUC of 0.89. Chan et al. used three CNNs (InceptionV3, ResNet101, and VGG19) to classify benign and malignant thyroid tumors, and the AUCs for the three CNNs were 0.82, 0.83, and 0.83, respectively.^41^^,^^42^

Until now, few studies have examined the application of AI in the classification of HCE subtypes. Liu et al. constructed a LeNet-5-architecture-based model to classify CE1 and CE2 from computed tomographic images with 97.5% accuracy.^43^ In the present study, we investigated the value of the AI in the classification of HCE. We used a VGG19-based CNN model (a multilayer small convolution kernel stack that includes 16 convolution layers and three fully connected layers and is useful in the extraction of microscopic visual features) for the classification of HCE subtypes from ultrasound images. The VGG19-based model showed excellent performance in the classification of CE1, CE2, CE4, and CE5, with precision and recall rates >0.85 and F1-scores >0.90. Moreover, the AI classifier more accurately identified the HCE subtypes than the use of manual classification by resident physicians did (90.4% and 86.1%, respectively; *P* <0.05).

In our study, the subtypes that were susceptible to misclassification and the corresponding misclassification ratios were similar to those of the AI classification and manual classification by the residents. Some sonographic features may have been responsible for the erroneous classifications. First, CE1 was misclassified as CE3. In some cases of CE1, there were likely to be “hydatid sand” in a single cyst, which was likely mistakenly identified as a curled inner capsule, or there were artifacts caused by reverberation and side lobe artifacts, which could have been identified as a collapsed inner capsule in static images. Second, CE2 was misclassified: 1) Subtype CE2 was misclassified as CE3. The typical sonographic features of CE2 is the “cyst in the cyst” sign, in which there are multiple spherical shadows and halos in the anechoic area of the parental cyst. In CE3, a collapsed and ruptured capsule floats in the anechoic fluid and is apparent as a “water lily” sign or “drift belt” sign. The occurrence of the high echoes in an anechoic area are similar to a feature of CE2. Therefore, CE2 was likely to be misidentified as CE3. 2) Subtype CE2 was misclassified as CE4. Long-term CE2 hydatid degeneration gradually occurred. One part was an active daughter cyst, and the other part was a daughter cyst with a wall that was folded and contracted. This gradually becomes necrotic and dissolves, and the cyst fluid is absorbed and concentrated to form a paste. Alternatively, caseous degeneration occurs, which manifests as heterogeneous hypoechoic areas or areas of hyperechoic degeneration. Moreover, AI failed to identify small, scattered daughter cysts and only identified larger areas of consolidated degeneration, thus suggesting necrotic consolidation. Third, subtype CE3 was misclassified as CE4. The internal capsule collapsed and peeled off, and the anechoic cystic fluid in the cyst was reduced. If the cystic fluid is not captured and if only the folded and contracted cyst wall is captured, CE3 is likely to be misclassified as CE4. Fourth, subtype CE4 was misclassified as CE5. In some cases of CE4, the lesion density is high, and the sound behind the lesion is attenuated; thus, this may be mistakenly identified as the wide sound shadow of CE5-type lesions. Fifth, subtype CE5 was misclassified as CE4. Some CE5 cysts had weak posterior sound shadows and were misclassified as CE4. The most frequent misclassification of subtype CE1 was CE3, which was made by both residents and AI, whereas the second most frequent misclassification of subtype CE1 was CE2 (30.2%) by residents and CE5 (15.1%) by AI. The residents seldom misclassified CE1 as CE5 (1.6%). We speculate that the residents may have mistakenly identified the reverberation artifacts as small daughter cysts, consequently misclassifying CE1 as CE2 (polycystic type). However, the residents could clearly distinguish the features of CE1, such as a hyperechoic cyst wall, anechoic cyst fluid, and enhanced echoes behind the lesion. They were unlikely to misjudge the anechoic cyst fluid as a wide acoustic shadow behind the calcification; therefore, the misclassification ratio of CE1 as CE5 by the residents was low.

There was no difference between the sensitivity of AI and resident physicians in terms of their recognition of treatment-required HCE (CE1–CE3; both 95.5%). However, the AI model more accurately identified treatment-free HCE (CE4 and CE5) than did the resident physicians (*P* <0.05). Therefore, the AI effectively reduced the number of patients who were misclassified as having treatment-free HCE, which reduced the risk of disease progression in misclassified patients who were prescribed follow-up observations rather than active treatment.

Our study suggests that AI performs better in the classification of HCE than manual classification by physicians and shows good potential for HCE classification and diagnosis. The application for the classification of HCE based on VGG19 is simple to operate; we are now developing an online application to make it more accessible to radiologists. This will benefit patients living in areas with scarce medical resources. However, the accuracy of the AI in HCE classification was not 100%. In particular, AI showed suboptimal recognition of CE3, identifying CE1 and CE2 as CE3. We do not know which image features are important for classification when AI identifies HCE US images. However, we can improve the performance of AI classification by increasing the volume of training data, adjusting the distribution of the training data, and optimizing the network structure—specifically, increasing the amount of CE3 the training model. In clinical practice, questionable US images still require manual inspection by a senior radiologist.

There are several limitations to our study. All the images in the training, validation, and test sets were collected from the First Affiliated Hospital of Xinjiang Medical University, which may have led to a less representative sample population. Moreover, our study classified only HCE subtypes and did not compare HCE with other liver-occupying lesions.

## CONCLUSION

The AI software that we developed based on the VGG19 algorithm more accurately (90.4%) classified HCE subtypes than manual classification by resident physicians (86.1%), suggesting that CNNs can assist physicians in classifying HCE subtypes and guide the choice of treatment. We expect that AI will provide a valuable alternative reference for radiologists, especially for those with little experience in the diagnosis of HCE subtypes. Insofar as AI avoids the subjective variability caused by manual reading, simple human errors are reduced, imaging physicians are able to subtype HCE quickly, and the efficiency and accuracy of HCE classification are improved.

## Supplemental Materials

10.4269/ajtmh.23-0519 Supplemental Materials
