# Supplementary material for: Clinical Application of Artificial Intelligence in the Ultrasound Classification of Hepatic Cystic Echinococcosis
Source: Am J Trop Med Hyg. 2024 May 28;111(1):93–101. doi: 10.4269/ajtmh.23-0519 (PMC11229658; doi:10.4269/ajtmh.23-0519)
Supplement: Supplemental Materials [file tpmd230519.SD1.pdf]

Automatic Classification of Hepatic Cystic Echinococcosis Using Ultrasound Images and Deep Learning

Miao Wu, MSc, Chuanbo Yan, MSc, Xiaorong Wang, MD, Qian Liu, MMed, Zhihua Liu, MMed, Tao Song, PhD

Received December 29, 2020, from the College of Biomedical Engineering and Instrumental Science, Zhejiang University, Hang Zhou, China (M.W.); College of Medical Engineering and Technology, Xinjiang Medical University, Urumqi, China (M.W., C.Y., Z.L.); Ultrasonography Department, First Affiliated Hospital of Xinjiang Medical University, Urumqi, China (X.W., T.S.); and Basic Medical College, Xinjiang Medical University, Urumqi, China (Q.L.). Manuscript accepted for publication February 24, 2021.

This work was supported by the Natural Science Foundation of China (81560294, 81760330, 81460281). The authors declare that they have no conflicts of interest.

Chuanbo Yan and Tao Song devised and supervised the project. Xiaorong Wang collected and verified data. Miao Wu and Qian Liu performed the computations and analyzed the results. Zhihua Liu solved some detailed problems in the experiment. Miao Wu proposed the methods and wrote the paper.

Ethical approval was obtained from the Research Ethics Committee of the Xinjiang Medical University. Both written and verbal consent was obtained in accordance with the ethics committee. Written informed consent for the publication of their clinical details and clinical images was obtained from the patients. A copy of the consent form is available for review with the editor of the journal.

The datasets generated and/or analyzed during the current study are not publicly available as they contain identifiable and personal information but are available from the corresponding author on reasonable request.

Address correspondence to Tao Song, Doctor, PhD, Ultrasonography Department of The First Affiliated Hospital of Xinjiang Medical University, Urumqi 830011, China.

E-mail: doctorsongtao@163.com

doi:10.1002/jum.15691

Background—Hepatic cystic echinococcosis is the main form of hepatic echinococcosis, which is a life-threatening liver disease caused by parasites that requires a precise diagnosis and proper treatment.

Objective—This study focuses on the automatic classification system of five different subtypes of hepatic cystic echinococcosis based on ultrasound images and deep learning algorithms.

Methods—Three popular deep convolutional neural networks (VGG19, Inception-v3, and ResNet18) with and without pretrained weights were selected to test their performance on the classification task, and the experiments were followed by a 5-fold cross-validation process.

Results—A total of 1820 abdominal ultrasound images covering five subtypes of hepatic cystic echinococcosis from 967 patients were used in the study. The classification accuracy for the models with pretrained weights (fine-tuning) ranged from 88.2 to 90.6%. The best accuracy of 90.6% was obtained by VGG19. For comparison, the models without pretrained weights (from scratch) achieved a lower accuracy, ranging from 69.4 to 75.1%.

Conclusion—Deep convolutional neural networks with pretrained weights are capable of recognizing different subtypes of hepatic cystic echinococcosis from ultrasound images, which are expected to be applied in the computer-aided diagnosis systems in future work.

Key Words—automatic classification; deep learning; hepatic cystic echinococcosis; pretrained weights; scratch; ultrasound images

Human cystic echinococcosis, or hydatid cyst disease, is a zoonosis caused by the larval cestode Echincoccus granulosus. Hippocrates recognized hydatid disease over 2000 years ago, and the disease remains endemic today in sheep-raising areas of the world, including Africa, the Mediterranean region of Europe, the Middle East, Asia, South America, Australia, and New Zealand. Dogs are the definitive hosts for Echincoccus granulosus and sheep the major intermediate host (yaks, goats, and camels are other relevant intermediate hosts); man is only incidentally infected. The liver is the most frequent site for the cystic lesions seen in hydatid disease, followed by the lung, brain, and other viscera.[1] The two most important forms of human

© 2021 American Institute of Ultrasound in Medicine

echinococcosis are cystic echinococcosis (CE) and alveolar echinococcosis (AE) caused by infection at the metacestode stages of echinococcus granulosus and echinococcus multilocularis, respectively.[2] In China, both CE and AE are highly endemic over large areas of the northwestern provinces and autonomous regions.[3] Human CE cases are responsible nationally for more than 98% of infections in China, with AE disease being the cause of the remainder.[4] Hepatic CE (HCE) may result in a wide spectrum of clinical manifestations ranging from asymptomatic infection to severe, even fatal, disease.[5] In 2001, the World Health Organization (WHO) Working Group of Experts on Hydatidosis proposed an international classification of hepatic cysts based on ultrasound morphology correlated to the activity of the disease,[6,7] which grouped hepatic hydatid cysts into five major cyst types, that is, single cystic type (SC or CE1), polycystic type (PC or CE2), internal capsule collapse type (ICC or CE3), solid mass type (SM or CE4), and calcified type (CA or CE5). A progressive natural history of cyst development from CE1 to CE5 is also considered.[8] CE1 and CE2 are classified as active cysts, and CE4 and CE5 are inactive cysts. CE3 cysts are classified as transitional. Cysts of different stages show very different responses to various management options.[9–12] Four therapeutic approaches are available for HCE: surgery, percutaneous techniques, drug treatment (albendazole) of active and transitional cysts, and the so-called watch and wait approach for inactive and selected cases of transitional CE3 cysts. Puncture, aspiration, injection, and reaspiration with a scolicidal agent such as 95% ethanol is the most widely used percutaneous technique for echinococcal cysts.[13] An accurate diagnosis is a prerequisite for optimal therapy. However, the ultrasound appearance of the cyst may change over time, either spontaneously or in response to treatment, leading to the difficulty of the diagnosis.[12] Currently, the gold standard for assignment into the five ultrasound subcategories is microscopic examination by analyzing the appearance of the cyst's contents and wall.[14] For patients with the CE1 or CE2 subtype that need surgery, the gold-standard test can be implemented because the cysts can be removed from the patients for the test. However, for other patients who only need drug treatment without surgery (ie, CE3) or patients who just need the watch-and-wait strategy (ie, CE4 and CE5), the gold-standard test can be hardly implemented, and ultrasonic examination is necessary. In clinical practice, the diagnosis of HCE is primarily based on ultrasound imaging with manual visual inspection,[15] which needs a high degree of skill and concentration and is prone to operator bias. The subjectivity of diagnostic criteria to each sonographer may also result in a poor interobserver agreement. An alternative approach would be the use of the computer-aided diagnosis (CAD) system, which has been applied in various diseases in the past few decades and achieved outstanding performance in most cases.[16-20] Recently, deep learning with convolutional neural networks (CNNs) has been gaining attention with respect to pattern recognition of images and as an artificial intelligence strategy used in CAD systems as it has distinct advantages over traditional machine learning methods in providing an end-to-end feature extraction and efficient classification framework to free users from the troublesome handcrafted feature extraction.[21–24] Over the last decade, growing national demand for accurate diagnosis of HCE at the hospitals in the undeveloped regions in China has propelled us to find efficient methods to help doctors improve their diagnostic performance. We hypothesized that the use of CNNs would recognize the sonographic appearance of different subtypes of HCE. Therefore, the aim of the present study was to assess the performance of the CNNs on classifying the 5 different subtypes of HCE from their sonographic images and provide scientific evidence for the future HCE CAD system. To the best of our knowledge, this is the first work on automatic classification of HCE into five different subtypes by using deep learning and sonographic images.

Materials and Methods

Subject and Data

This study was approved by the Institutional Review Board of The First Affiliated Hospital of Xinjiang Medical University in China, and informed consent was obtained from the research subjects. The data were retrospectively obtained from The First Affiliated Hospital of Xinjiang Medical University between

Table 1. The Ultrasound Images and Patients' Number of Five Subtypes of Hepatic Cystic Echinococcosis (HCE) Involved in the Study

Subtypes of HCE	Number of Ultrasound Images	Number of Patients
Single cystic (SC)	358	174
Polycystic (PC)	383	194
Internal capsule collapse (ICC)	350	156
Calcified (CA)	351	213
Solid mass (SM)	378	230

the years 2008 and 2020. A total of 1820 abdominal ultrasound images covering the five different subtypes of HCE from 967 patients were involved in this study, including 358 images of 174 SC patients, 383 images of 194 PC patients, 350 images of 156 ICC patients, 351 images of 213 CA patients, and 378 images of 230 SM patients, which were shown in Table 1. To protect the patient's privacy, the personal information on the image has been removed, and Figure 1 shows samples of each subtype

Figure 1. Ultrasound image samples of each subtype of hepatic cystic echinococcosis: **A**, single cystic type, **B**, polycystic type, **C**, calcified type, **D**, internal capsule collapse type, and **E**, solid mass type.

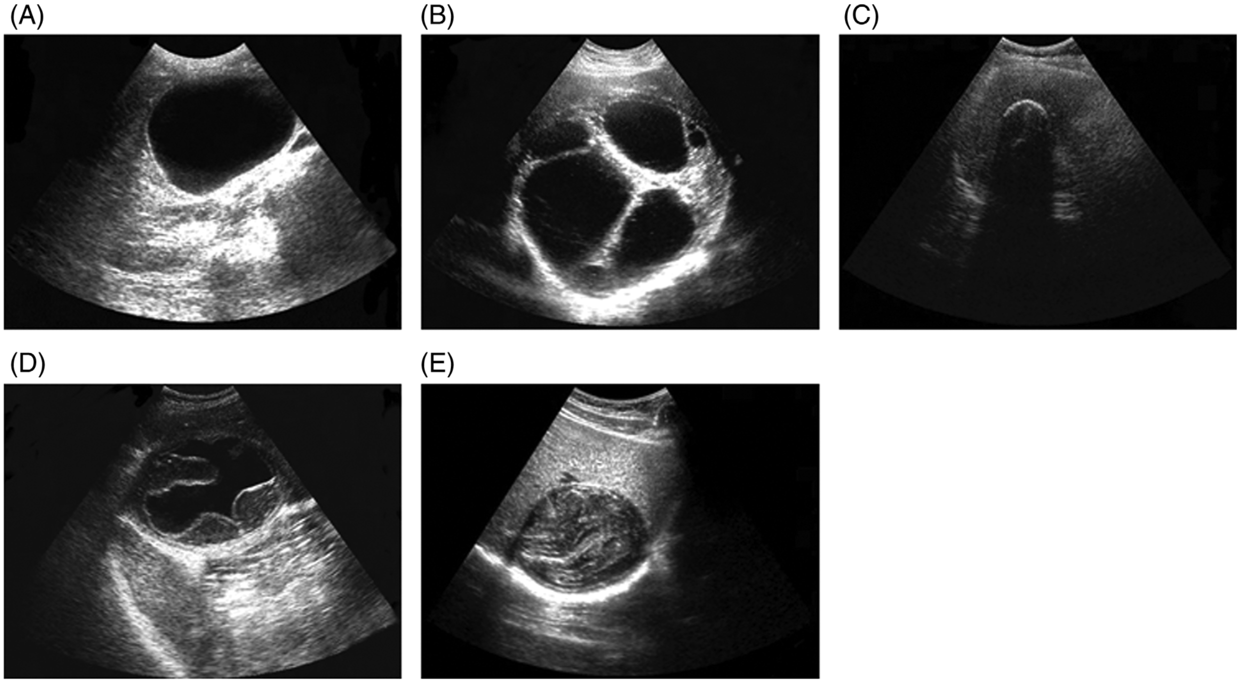

Figure 2. Removal and reparation of artificial markers in a hepatic cystic echinococcosis ultrasound image. **A**, Image with markers, **B**, repaired image

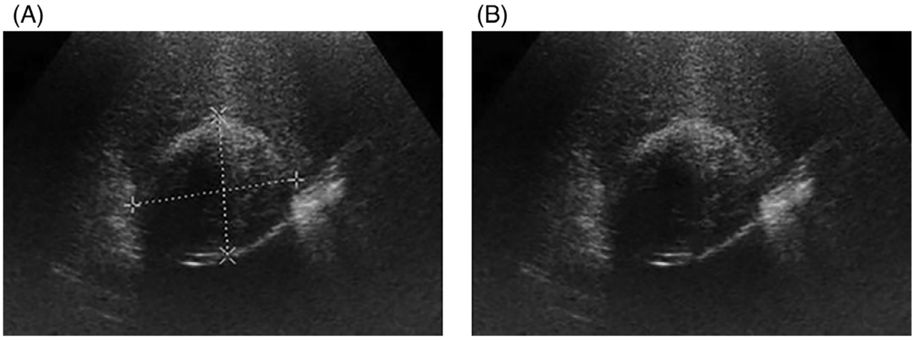

Figure 3. The flow of the study, starting with original ultrasound images followed by preprocessing to obtained qualified images for deep learning

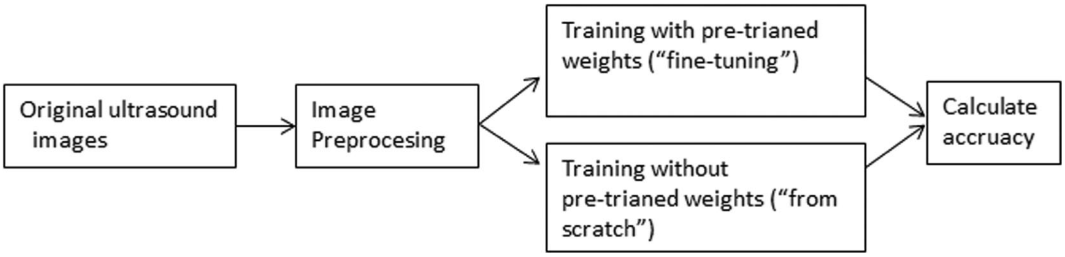

of HCE. All the images were clinically confirmed by at least two experienced sonographers.

Image Preprocessing

Before using the images to train and test the CNNs, image preprocessing is needed, including artificial marker repair and region of interest (ROI) extraction. A portion of these ultrasound images was artificially marked by sonographers to record the location and size of the cystic capsule, etc. As shown in Figure 2A, artificial markers occlude the texture region and break the integrity of an image, adversely affecting image analysis. Hence, we used Spot Healing Brush in Adobe Photoshop (version 19.1.1) to remove the markers and repair the image. In particular, we used a rounded Spot Healing Brush with a diameter of 15 pixels that was slightly larger than the width of the graticules and a hardness of 0% to avoid harsh and unnatural edges; other settings for the brush were spacing of 25%, angle of 0%, and roundness of 100%. Then, we use this Spot Healing Brush to remove the spots (markers) by brushing along the graticules. As shown in Figure 2B, the makers are entirely repaired with good image quality. This ensures that the training images have no artificially labeled or irrelevant information that may affect the classification results, enhancing the models' reliability.

The original images showed a large echo region, including the liver and other peripheral organ parts. To eliminate the interference of irrelevant areas and reduce the computational burden of the model, we manually cropped the lesion region as the ROI and set the size to a uniform 224 × 224 in the JPG format.

Methods

The experiments were implemented in MATLAB 2020b on a computer with specifications of 32GB RAM and Intel® Core™ i7-8700 @ 3.2GHz CPU. Figure 2 shows the flow of this study.

Table 2. Confusion Matrices by Fine-Tuning the Networks

(A) VGG19

	Predicted				
**Actual**	**CA**	**ICC**	**PC**	**SC**	**SM**
CA	335	0	1	3	12
ICC	0	287	37	17	9
PC	2	8	360	8	5
SC	3	5	14	336	0
SM	33	5	9	1	330

(B) Inception-v3

	Predicted				
**Actual**	**CA**	**ICC**	**PC**	**SC**	**SM**
CA	334	2	0	2	13
ICC	4	292	17	23	14
PC	5	20	329	17	12
SC	6	9	6	337	0
SM	20	7	2	2	347

(C) ResNet18

	Predicted				
**Actual**	**CA**	**ICC**	**PC**	**SC**	**SM**
CA	328	0	4	5	14
ICC	5	253	46	32	14
PC	3	7	343	24	6
SC	2	0	9	347	0
SM	26	3	9	6	334

Due to their outstanding performance in previous studies,[25–27] we choose the following network architectures as the classification models: VGG19,[28] Inception-v3,[29] and ResNet18.[30] For each architecture, the training processes were performed as

follows: (1) training the networks with random initial weights ("from scratch") by the preprocessed images and (2) training with publicly available pretrained networks ("fine-tuning") by the preprocessed images. The overall flow of analyses is summarized in Figure 3. The hyperparameters of the networks were heuristically adjusted so as to facilitate the convergence of the loss function during the training. Cross-entropy is the loss function used as it gives the measure of the closeness of the predicted and actual distribution. Stochastic gradient descent with momentum was the chosen optimizer, considering its good learning rate and the parameter-specific adaptive nature of the learning rates. The initial learning rate was set to 0.0003 as a high value might prevent the loss function from converging and cause overshoots, and a very small value increases the burden of the computation and training time. Considering the speed of training (a large batch size means faster training) and the computational capacity of the computer, the mini-batch size was set to 30. Besides, a very large batch size adversely impacts the models' performance. A higher learning rate is desirable at the modified fully connected layer so as to learn specific features of the ultrasound images, so the learning factor was set to 10. An epoch is equivalent to a forward pass and a backward pass of all the training examples. In our experiment, the number of epochs was limited to 10 considering the occurrence of overfitting. Owing to the limited data size in our study, the following methods of data augmentation were adopted during the training process of each model to reduce overfitting: horizontal flip, vertical flip, translation (±5 pixels along X-axis or Y-axis), zoom (from 0.9 to 1.1), and rotation (±5°).

Table 3. Confusion Matrices by Training From Scratch (Networks with Random Initial Weights)

(A) VGG19

	Predicted				
**Actual**	**CA**	**ICC**	**PC**	**SC**	**SM**
CA	291	1	7	13	39
ICC	4	230	48	46	22
PC	5	72	234	40	32
SC	17	22	10	306	3
SM	37	14	19	2	306

(B) Inception-v3

	Predicted				
**Actual**	**CA**	**ICC**	**PC**	**SC**	**SM**
CA	311	2	4	1	33
ICC	8	183	104	35	20
PC	5	105	209	26	38
SC	18	17	33	284	6
SM	59	16	26	1	276

(C) ResNet18

	Predicted				
**Actual**	**CA**	**ICC**	**PC**	**SC**	**SM**
CA	256	1	3	11	80
ICC	3	234	42	41	30
PC	5	87	215	28	48
SC	7	19	5	318	9
SM	24	8	9	2	335

To evaluate the models' performance, classification accuracy, recall, specificity, precision, and F1-score were calculated along with a confusion matrix. The following Equations (1)–(5) defined the metrics.

$$\text{Accuracy} = \frac{TP + TN}{TP + FP + TN + FN} \quad (1)$$

$$\text{Recall} = \frac{TP}{TP + FN} \quad (2)$$

$$\text{Specificity} = \frac{TN}{TN + FP} \quad (3)$$

$$\text{Precision} = \frac{TP}{TP + FP} \quad (4)$$

$$F1 - \text{score} = \frac{2 * \text{precision} * \text{recall}}{\text{precision} + \text{recall}} \quad (5)$$

where TP, FP, TN, and FN represent the numbers of true positive, false positives, true negatives, and false negatives, respectively. We performed the experiments five times, and each experiment followed a 5-fold cross-validation process. The average results after 5 trials were presented in mean ± standard deviation format.

Results

A confusion matrix is a tabular summary of the number of correct and incorrect predictions made by a classifier. Table 2 shows the confusion matrices by

fine-tuning the networks, and Table 3 shows the confusion matrices by training from scratch (networks with random initial weights), which were obtained during experimentation. Based on the confusion matrices, different metrics can be derived to indicate the models' performance, specific to each HCE subtype class as shown in Table 4. The overall classification accuracy from high to low was 90.6 ± 1.3% for fine-tuning VGG19, 90.1 ± 1.6% for fine-tuning Inception-v3, 88.2 ± 1.5% for

Table 4. Hepatic Cystic Echinococcosis (HEC) Class-Specific Evaluation of Fine-Tuning the Networks

(A) VGG19

HCE Subtypes	Precision (%)	Recall (%)	Specificity (%)	F1-Score (%)	AUC (%)
Calcified type (CA)	89.9 ± 3.0	95.4 ± 2.1	97.2 ± 1.0	92.6 ± 1.3	99.7 ± 0.2
Internal capsule collapse type (ICC)	94.2 ± 2.3	82.0 ± 4.6	98.7 ± 0.6	87.6 ± 2.4	98.5 ± 0.7
Polycystic type (PC)	85.8 ± 5.0	94.0 ± 2.0	95.5 ± 1.8	89.6 ± 2.8	98.4 ± 0.6
Single cystic type (SC)	92.5 ± 4.9	94.0 ± 3.8	97.8 ± 1.5	93.0 ± 1.4	99.4 ± 0.3
Solid mass type (SM)	92.8 ± 2.6	87.3 ± 6.4	98.1 ± 0.8	89.8 ± 3.5	99.0 ± 0.4

(B) Inception-v3

HCE Subtypes	Precision (%)	Recall (%)	Specificity (%)	F1-Score (%)	AUC (%)
CA	91.0 ± 5.4	95.2 ± 3.2	97.4 ± 1.7	92.8 ± 2.0	99.5 ± 0.3
ICC	89.9 ± 7.6	83.4 ± 10.3	97.3 ± 2.4	85.7 ± 4.5	98.7 ± 0.2
PC	93.0 ± 2.3	85.9 ± 2.7	98.1 ± 0.7	89.3 ± 2.2	98.6 ± 0.5
SC	89.0 ± 5.7	94.2 ± 3.9	96.7 ± 1.9	91.3 ± 1.1	99.4 ± 0.2
SM	90.7 ± 6.7	91.8 ± 4.9	97.1 ± 2.4	90.9 ± 2.5	98.8 ± 0.5

(C) ResNet18

HCE subtypes	Precision (%)	Recall (%)	Specificity (%)	F1-Score (%)	AUC (%)
CA	91.0 ± 7.5	93.5 ± 5.2	97.3 ± 2.5	91.9 ± 3.0	99.5 ± 0.3
ICC	96.2 ± 1.4	72.3 ± 4.7	99.3 ± 0.2	82.5 ± 3.3	98.5 ± 0.6
PC	83.9 ± 5.4	89.6 ± 3.6	94.9 ± 2.1	86.5 ± 2.0	98.4 ± 0.7
SC	84.0 ± 3.4	96.9 ± 1.6	94.9 ± 1.3	89.9 ± 1.7	99.4 ± 0.3
SM	91.8 ± 7.1	88.4 ± 6.9	97.4 ± 2.4	89.6 ± 2.0	98.9 ± 0.4

Figure 4. The overall classification accuracy of each convolutional neural network in our experiment. ('FT' is short for 'fine-tuning,' and 'S' is short for 'scratch')

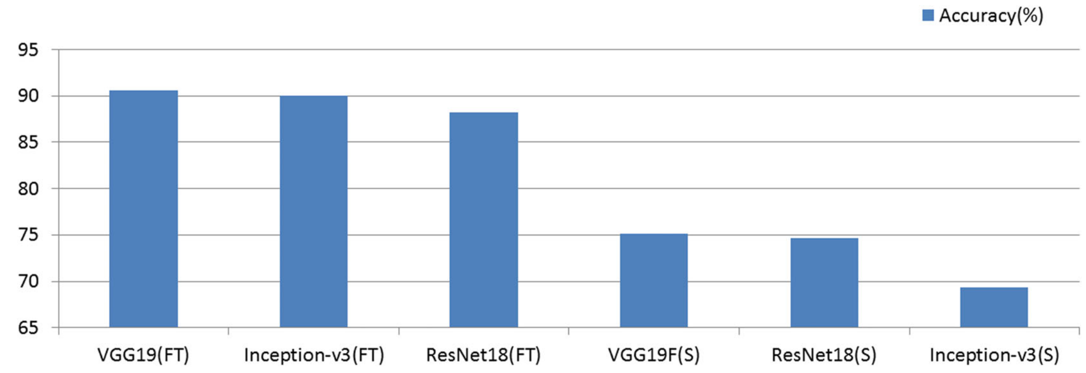

fine-tuning ResNet18, 75.1 ± 3.9% for VGG19 from scratch, 74.6 ± 1.4% for ResNet from scratch, and 69.4 ± 3.3% for Inception-v3 from scratch, as shown in Figure 4.

Figures 5 and 6 demonstrate the accuracy of training and validation datasets by the three CNNs with and without pretrained weights, respectively.

Figure 5. Accuracy of the training and validation datasets by the three convolutional neural networks. Pretrained model weights were used for training ("fine-tuning"). **A**, VGG19; **B**, Inception-v3, and **C**, ResNet18

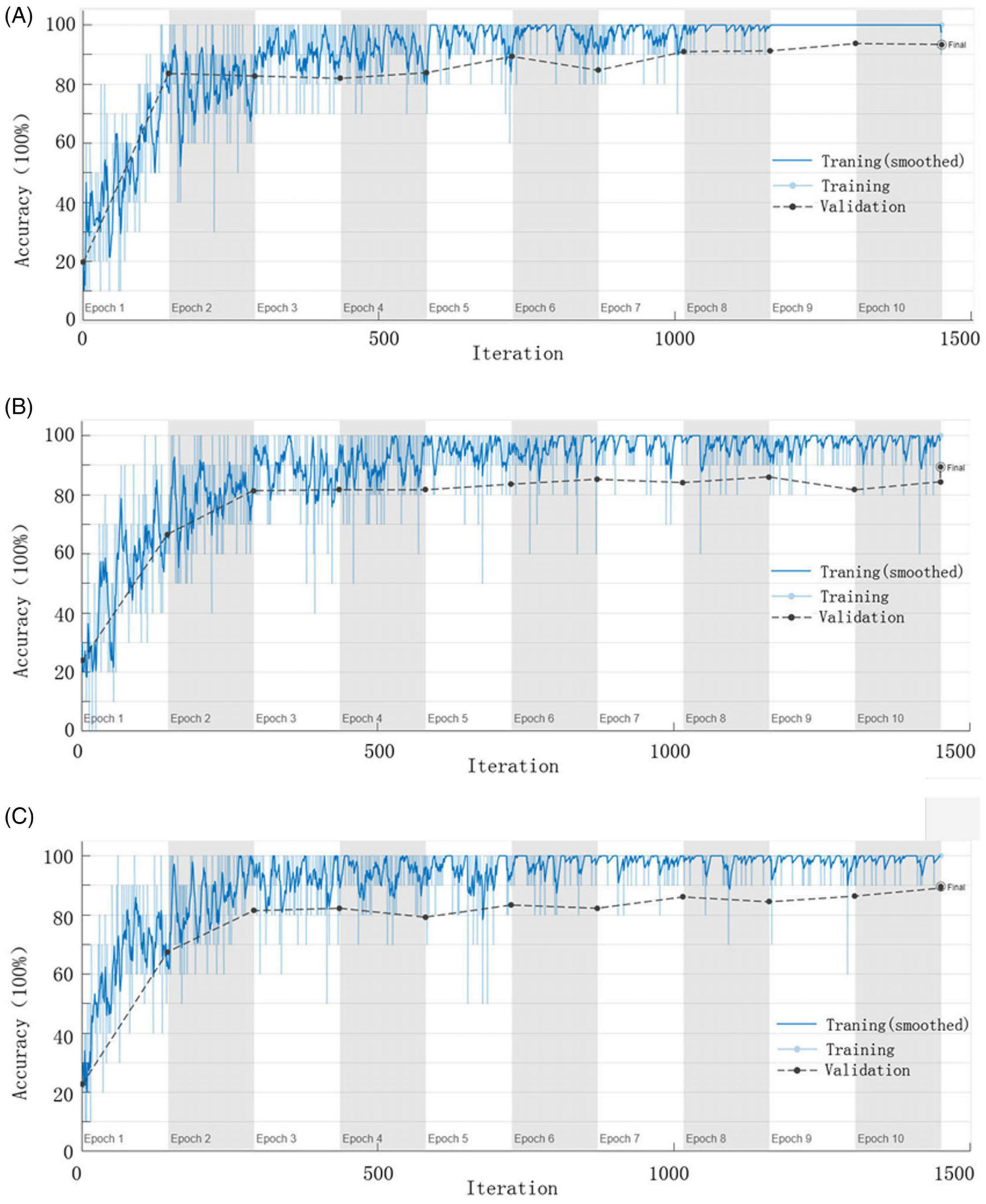

Figure 6. Accuracy of the training and validation datasets by the three convolutional neural networks. The model weights used random initial weights ("training from scratch"): **A**, VGG19; **B**, Inception-v3, and **C**, ResNet18

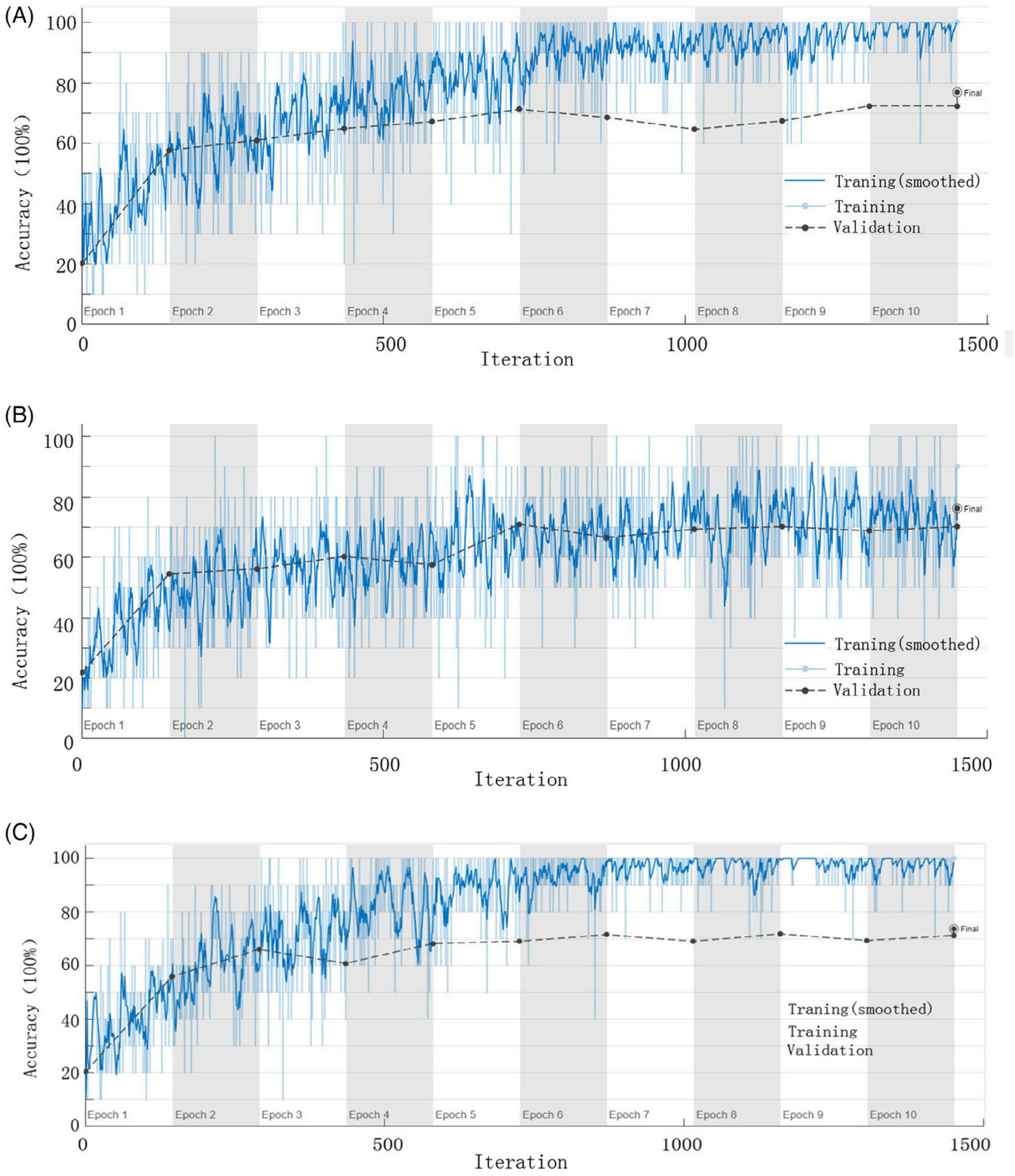

As the fine-tuning VGG19 obtained the best accuracy in our experiments, we illustrate its receiver operating characteristic (ROC) curve in Figure 7 to demonstrate its classification ability, and the corresponding values of the Area Under the Curve (AUC) are listed in the Table 2.

Discussion

In this study, we evaluated the performance of deep learning algorithms in the automatic classification of five different subtypes of HCE from ultrasound images based on the 5-fold cross-validation. The average

classification accuracy ranged from 88.2 to 90.6% for the fine-tuning networks. The best performance was achieved by the fine-tuning VGG19 with a classification accuracy of 90.6 ± 1.3%. For comparison, similar experiments were performed without the use of pretrained weights ("training from scratch") that showed poorer performance (Table 3 and Figure 6), and average classification accuracy only ranged from 69.4 to 75.1%. A T-test indicated there was a statistically significant difference between the classification accuracy for each model training with pretrained weights and the model from scratch training with random initial weights ($P < .001$). In addition to the classification accuracy, other evaluation metrics shown in Tables 4 and 5 also verified that the performance of fine-tuning networks is superior to those networks from scratch.

Figure 7. Receiver operating characteristic curves of VGG19 with pretrained weights

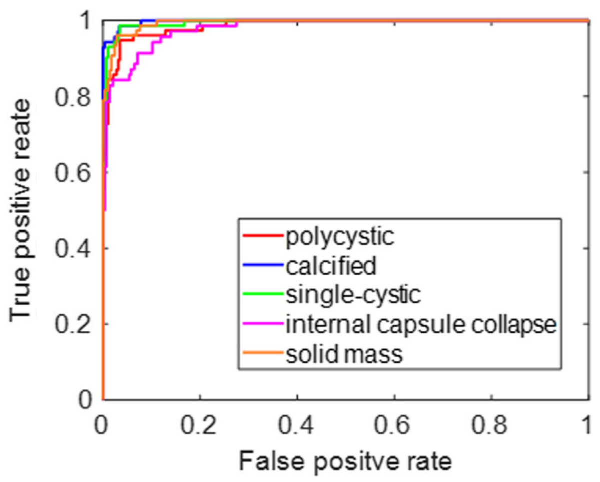

A small training dataset can be a weakness in deep learning algorithms because it can be easily overfitted.[31] To overcome the limitations, the techniques of data augmentation and transfer learning were often used in many medical fields, such as computerized tomography (CT), magnetic resonance imaging (MRI), and ultrasound images,[32-35] which have been proven to be efficient ways for improving the performance of deep learning models. Since the

Table 5. Hepatic Cystic Echinococcosis (HCE) Class-Specific Evaluation of the Networks from Scratch

(A) VGG19

HCE Subtypes	Precision (%)	Recall (%)	Specificity (%)	F1-Score (%)	AUC (%)
Calcified type (CA)	82.6 ± 2.8	82.9 ± 12.2	94.5 ± 1.6	82.1 ± 5.8	97.8 ± 0.8
Internal capsule collapse type (ICC)	70.0 ± 9.6	65.7 ± 8.7	91.2 ± 4.6	66.7 ± 4.2	90.4 ± 3.4
Polycystic type (PC)	76.1 ± 8.2	61.1 ± 18.8	93.2 ± 4.0	65.0 ± 11.8	91.8 ± 1.5
Single cystic type (SC)	78.0 ± 10.8	85.4 ± 11.0	91.3 ± 7.1	80.3 ± 6.6	97.7 ± 0.4
Solid mass type (SM)	76.5 ± 3.2	81.0 ± 9.0	91.6 ± 2.3	78.3 ± 3.5	96.2 ± 0.6

(B) Inception-v3

HCE Subtypes	Precision (%)	Recall (%)	Specificity (%)	F1-Score (%)	AUC (%)
CA	79.2 ± 9.0	88.6 ± 6.9	91.3 ± 5.4	82.9 ± 2.9	98.0 ± 0.3
ICC	59.7 ± 7.4	52.3 ± 26.8	88.8 ± 7.0	50.3 ± 15.8	86.4 ± 3.5
PC	57.8 ± 6.7	54.6 ± 14.0	86.2 ± 7.4	54.4 ± 3.9	86.7 ± 0.9
SC	84.1 ± 9.9	79.3 ± 9.1	94.0 ± 4.8	80.6 ± 4.1	96.5 ± 0.9
SM	76.1 ± 7.9	73.0 ± 17.4	91.1 ± 5.2	72.5 ± 8.9	95.4 ± 1.0

(C) ResNet18

HCE Subtypes	Precision (%)	Recall (%)	Specificity (%)	F1-Score (%)	AUC (%)
CA	88.5 ± 7.5	72.9 ± 12.0	96.6 ± 2.9	78.8 ± 5.6	98.1 ± 0.3
ICC	68.2 ± 7.4	66.9 ± 8.6	90.8 ± 3.3	66.8 ± 3.4	91.3 ± 1.7
PC	78.7 ± 5.9	56.1 ± 8.6	95.1 ± 1.8	65.1 ± 6.8	92.5 ± 1.2
SC	80.4 ± 6.2	88.9 ± 7.2	92.7 ± 3.1	83. 9 ± 2.0	97.6 ± 0.5
SM	68.4 ± 8.5	88.6 ± 6.6	86.2 ± 5.9	76.5 ± 3.9	96.4 ± 0.7

Table 6. The Fine-Tuning Models' Predictions

Sample Images	Actual	ResNet18	Inception-v3	VGG19
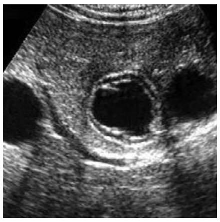	SC	PC	SC	SC
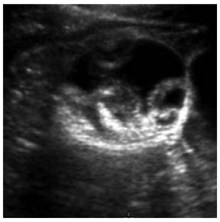	PC	PC	PC	ICC
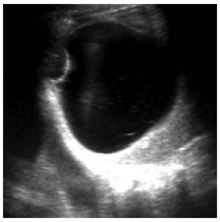	ICC	SC	ICC	ICC
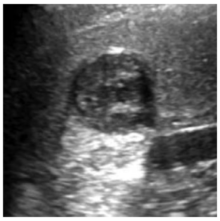	SM	ICC	SM	SM
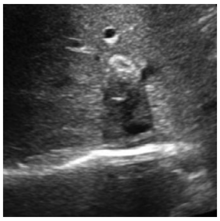	CA	SM	CA	SM

available data in our experiments is limited, both of these two methods were applied in our experiments. With the help of data augmentation, the size of the training dataset can be artificially expanded from the exiting images by image flip, rotation, translation, etc. to reduce the risk of overfitting caused by the lack of

available training data.[36] We applied data augmentation for the training data with random parameter values within specified ranges. As for transfer learning, also known as knowledge transfer, it is a machine learning method that reuses a pretrained model on a new problem, which exploits the knowledge gained from a previous task to improve generalization about another.[37] We used the pretrained CNNs with publicly available weights that were optimized by 1.2 million general-purpose images in 1000 classes on ImageNet.[38] Although ImageNet did not include medical images, the pretrained CNNs were capable of extracting valuable characteristics of many other fields of images for recognition. As for our experiments, confusion matrices in Table 2 and the training process shown in Figure 5 both indicate that the pretrained CNNs correctly classified most of the images. However, there were still some misclassified image samples in the results. It is interesting to note that, for the same testing images, the classification results of the three pretrained models are not completely consistent due to their different architecture and features extracted. As shown in Table 6, some of the images misclassified by a pretrained model were correctly classified by the others. This finding inspires us to use an ensemble of CNNs that comprehensively considers the results of three CNNs model to further enhance the classification accuracy.

As this is a pilot study for automatic classification of HCE by deep CNNs, some limitations exist. One is that we manually cropped ROIs from the original ultrasound images to form the training and testing dataset, which may be subjective and could prevent the system from becoming fully automatic. The automatic methods of image segmentation, ie, U-Net,[39] SegNet,[40] etc. are expected to be applied and assessed in future studies. Another shortage is that the generalizability of the models was unknown as all image data were collected from a hospital. Future prospective multicenter studies in a larger cohort of patients are needed to validate the models' generalizability.

Conclusion

This study demonstrated that CNNs are capable of recognizing different subtypes of HCE from ultrasound images. The fine-tuning CNNs with pretrained weights outperform the CNNs from scratch. The best performance was achieved by the fine-tuning VGG19 with pretrained weights, which achieved an overall accuracy of 90.6%. Our work suggested that CNNs can be important methods in the HCE CAD system and could help clinicians improve their diagnostic performance.

References

1. Chin J. *Echinococcosis, Control of communicable diseases manual.* Washington, DC: American Public Health Association; 2000; 176–179.
2. Feng X, Qi X, Yang L, et al. Human cystic and alveolar echinococcosis in the Tibet Autonomous Region (TAR), China. *J Helminthol* 2015; 89:671–679.
3. Philip SC, Disease H. Epidemiology of human alveolar echinococcosis in China. *Parasitol Int* 2006; 221–225.
4. Craig PS. The Echinococcosis working Group in C, Echinococcosis working Group in C. Epidemiology of human alveolar echinococcosis in China. *Parasitol Int* 2006; 55:S221–S225.
5. Brunetti E, Tamarozzi F, Macpherson C, et al. Ultrasound and cystic echinococcosis. *Ultrasound Int Open* 2018; 4:E70–E78.
6. Gharbi HA, Hassine W, Brauner MW, Dupuch K. Ultrasound examination of the hydatic liver. *Radiology* 1981; 139:459–463.
7. Brunetti E, Kern P, Vuitton DA, Writing Panel W-I. Expert consensus for the diagnosis and treatment of cystic and alveolar echinococcosis in humans. *Acta Tropica* 2010; 114:1–16.
8. Siracusano A, Teggi A, Ortona E. Human cystic echinococcosis: old problems and new perspectives. *Interdiscip Perspect Infect Dis* 2009; 2009:474367–474368.
9. Stojkovic M, Zwahlen M, Teggi A, et al. Treatment response of cystic echinococcosis to benzimidazoles: a systematic review. *PLoS Neglect Trop Dis* 2009; 3:e524.
10. Rinaldi F, De Silvestri A, Tamarozzi F, Cattaneo F, Lissandrin R, Brunetti E. Medical treatment versus "watch and wait" in the clinical management of CE3b echinococcal cysts of the liver. *BMC Infect Dis* 2014; 14:492–492.
11. Piccoli L, Tamarozzi F, Cattaneo F, et al. Long-term sonographic and serological follow-up of inactive echinococcal cysts of the liver: hints for a "watch-and-wait" approach. *PLoS Neglect Trop Dis* 2014; 8:e3057.
12. Golemanov B, Grigorov N, Mitova R, et al. Efficacy and safety of PAIR for cystic echinococcosis: experience on a large series of patients from Bulgaria. *Am J Trop Med Hygiene* 2011; 84:48–51.
13. Dehkordi AB, Sanei B, Yousefi M, et al. Albendazole and treatment of hydatid cyst: review of the literature. *Infect Disord Drug Targets* 2019; 19:101–104.

14. Varedi P, Saadat Mostafavi SR, Salouti R, et al. Hydatidosis of the pelvic cavity: a big masquerade. *Infect Dis Obstet Gynecol* 2008; 2008:782621.
15. Tamarozzi F, Covini I, Mariconti M, et al. Comparison of the diagnostic accuracy of three rapid tests for the serodiagnosis of hepatic cystic echinococcosis in humans. *PLoS Neglect Trop Dis* 2016; 10: e0004444.
16. Asiri N, Hussain M, Al Adel F, Alzaidi N. Deep learning based computer-aided diagnosis systems for diabetic retinopathy: a survey. *Artif Intell Med* 2019; 99:101701.
17. Ahmadi A, Kashefi M, Shahrokhi H, Nazari MA. Computer aided diagnosis system using deep convolutional neural networks for ADHD subtypes. *Biomed Signal Process Control* 2021; 63:102227.
18. Bai ZY, Chang LC, Yu RG, et al. Thyroid nodules risk stratification through deep learning based on ultrasound images. *Med Phys* 2020; 47:6355–6365.
19. Guo LJ, Gong H, Wang QS, et al. Detection of multiple lesions of gastrointestinal tract for endoscopy using artificial intelligence model: a pilot study. *Surg Endosc Other Interv Tech* 2020.
20. Lbachir IA, Daoudi I, Tallal S. Automatic computer-aided diagnosis system for mass detection and classification in mammography. *Multimedia Tools Appl* 2021; 80:9493–9525.
21. Han Y, Kim D. Deep convolutional neural networks for pan-specific peptide-MHC class I binding prediction. *BMC Bioinform* 2017; 18:585.
22. Jia HZ, Xia Y, Song Y, et al. 3D APA-net: 3D adversarial pyramid anisotropic convolutional network for prostate segmentation in MR images. *IEEE Trans Med Imaging* 2020; 39:447–457.
23. Shoeibi A, Ghassemi N, Alizadehsani R, et al. A comprehensive comparison of handcrafted features and convolutional autoencoders for epileptic seizures detection in EEG signals. *Expert Syst Appl* 2021; 163:113788.
24. Yang ZB, Ran LY, Zhang SZ, Xia Y, Zhang YN. EMS-net: ensemble of multiscale convolutional neural networks for classification of breast cancer histology images. *Neurocomputing* 2019; 366:46–53.
25. Jain R, Nagrath P, Kataria G, Kaushik VS, Hemanth DJ. Pneumonia detection in chest X-ray images using convolutional neural networks and transfer learning. *Measurement* 2020; 165:108046.
26. Liu ZY, Yang C, Huang J, Liu SP, Zhuo YM, Lu X. Deep learning framework based on integration of S-mask R-CNN and inception-v3 for ultrasound image-aided diagnosis of prostate cancer. *Future Gener Comput Syst Int J Esci* 2021; 114:358–367.
27. Ahuja S, Panigrahi BK, Dey N, Rajinikanth V, Gandhi TK. Deep transfer learning-based automated detection of COVID-19 from lung CT scan slices. *Appl Intell* 2021; 51:571–585.
28. Simonyan K, Zisserman A. Very deep convolutional networks for large-scale image recognition. *arXiv*; 2014.
29. Szegedy C, Liu W, Jia Y, et al. Going deeper with convolutions. *IEEE Conference on Computer Vision and Pattern Recognition* 2015; 1–9.
30. He K, Zhang X, Ren S, Sun J. Deep residual learning for image recognition. *IEEE Conference on Computer Vision and Pattern Recognition* 2016; 770–778.
31. Nodera H, Takamatsu N, Yamazaki H, et al. Deep learning for identification of fasciculation from muscle ultrasound images. *Neurol Clin Neurosci* 2019; 7:267–275.
32. Yang Q, Wei JW, Hao XH, et al. Improving B-mode ultrasound diagnostic performance for focal liver lesions using deep learning: a multicentre study. *Ebiomedicine* 2020; 56:102777.
33. Wang J, Zhu HD, Wang SH, Zhang YD. A review of deep learning on medical image analysis. *Mobile Netw Appl* 2020.
34. Xi IL, Wu J, Guan J, et al. Deep learning for differentiation of benign and malignant solid liver lesions on ultrasonography. *Abdom Radiol* 2021; 46:534–543.
35. Zheng J, Lin DA, Gao ZJ, Wang S, He MJ, Fan JP. Deep learning assisted efficient AdaBoost algorithm for breast cancer detection and early diagnosis. *IEEE Access* 2020; 8:96946–96954.
36. Mi Q, Xiao Y, Cai Z, Jia XB. The effectiveness of data augmentation in code readability classification. *Inf Softw Technol* 2021; 129:106378.
37. Alencastre-Miranda M, Johnson RR, Krebs HI. Convolutional neural networks and transfer learning for quality inspection of different sugarcane varieties. *IEEE Trans Ind Inform* 2020; 17:787.
38. Russakovsky O, Deng J, Su H, et al. ImageNet large scale visual recognition challenge. *Int J Comput Vis* 2015; 115:211–252.
39. Ronneberger O, Fischer P, Brox T. U-Net: Convolutional Networks for Biomedical Image Segmentation. In: Navab N., Hornegger J., Wells W., Frangi A. (eds) *Medical Image Computing and Computer-Assisted Intervention – MICCAI 2015*. Cham, Switzerland: Springer; 2015.
40. Badrinarayanan V, Kendall A, Cipolla R. SegNet: a deep convolutional encoder-decoder architecture for image segmentation. *IEEE Trans Pattern Anal Machine Intell* 2019; 39:2481–2495.
